# Analysis of the Influence of External Wall Material Type on the Thermal Bridge at the Window-to-Wall Interface

**DOI:** 10.3390/ma16196585

**Published:** 2023-10-06

**Authors:** Janina Adamus, Marta Pomada

**Affiliations:** Department of Civil Engineering, Faculty of Civil Engineering, Czestochowa University of Technology, 69 Dabrowskiego St., 42-201 Czestochowa, Poland; pomada.marta@gmail.com

**Keywords:** construction materials, sustainable building materials, sustainable development, energy saving, energy efficiency, composite beams, window installation, building application

## Abstract

Background: Although many works focus on increasing the energy efficiency of buildings, there are still a number of problems that need to be solved, such as reducing heat losses at the window-to-wall interface, especially since the requirements for saving energy used for heating/cooling rooms are constantly increasing. This paper analyses the impact of the material parameters of the external wall and the window installation in the insulation layer on the size of thermal bridges around the window. Purpose: The aim of the work is to demonstrate the benefits resulting from the correct installation of the window, the appropriate location of the window in relation to the face of the external wall, as well as the correct selection of construction materials. Methodology: In order to show the improvement in the energy efficiency of buildings, an analysis of the heating/cooling energy consumption was carried out for the selected buildings. The thermal and humidity analyses were carried out using TRISCO program, while the economic analysis was performed using the Audytor OZC program. Results: It was found that the proposed system of window installation in the thermal insulation layer reduced the annual heating demand by at least 10% on average. Conclusions: It has been shown that the method of window installation and the type of the wall structural materials are interrelated and therefore should be considered simultaneously. Their proper selection allows for a reduction in the amount of energy needed for heating and cooling buildings, and thus a reduction of heating/cooling costs, as well as limiting greenhouse gas emissions.

## 1. Introduction

According to the report of the International Energy Agency (IEA) [1], the building sector was responsible for 132 EJ of energy consumption in 2021, or 30% of total global final energy consumption and 27% of total energy sector emissions. Similar data provided by the European Commission report [2] from 2021 shows that in 2019, households in the EU accounted for 26.3% of final energy consumption and 11.3% of total CO_2_ emissions. Although in the EU, energy consumption in households per 1 m^2^ shows a slight downward trend (in 2019, the amount of energy consumption was 16.5 kgoe/m^2^, which is a 2.5% decrease compared to 21.3 kgoe/m^2^ in 2009 [3]), along with the growing energy crisis and the rapid increase in energy costs, the need to build energy-efficient buildings, both residential and public, is becoming more and more important. The most burdensome component of energy use is space heating, whose share in 2019 was 63.2%, and as emphasized by Khouki [4], in countries characterized by extremely hot or cold climate, this consumption increases to 70%. The resulting greenhouse gas emissions are the cause of climate change, which is why reduction of gas emissions is becoming the most pressing issue of our time. The authors of papers [5,6] emphasize that in order to reduce energy consumption and carbon dioxide emissions, it is necessary not only to increase the share of renewable energy in global energy systems but also to change consumer behaviour in order to reduce energy waste. Therefore, national governments, municipalities, and companies are setting net-zero emissions goals. More and more countries are declaring their readiness to fulfil such commitments in the coming decades. But even if government commitments were fully met, according to the authors of paper [7], it will still be far from the assumption that in 2050 carbon dioxide emissions related to global energy will fall to net zero, which could limit the global temperature increase to 1.5 °C. Achieving such a level would require the mass implementation of all available clean energy technologies, including renewable energy sources, electric vehicles, and energy-efficient buildings, especially since, as noted in [8], buildings are becoming the largest energy “consumers” in the world. This is because most people spend 90% of their time indoors every day and, to improve their living conditions, are consuming more and more energy on heating–ventilation–air conditioning (HVAC) systems, hot water production systems, lighting systems, and other outdoor equipment.

In March 2023, the European Parliament implemented changes to the Energy Performance of Buildings Directive (EPBD) [9], including the necessity of the GWP (Global Warming Potential) introduction to the energy performance of buildings. It defines the CO_2_e/m² emission equivalent (usable area) in kg, summed up for each stage of the technical life cycle of the building and averaged over one reference year over the 50-year study period. These requirements apply not only to newly designed and erected buildings, but also to existing buildings undergoing modernization. Therefore, it will be necessary to use technological solutions and structural materials that will not only reduce the operating costs of buildings but will also reduce the value of the GWP of the analysed object. The EPBD requires EU countries to ensure that all new buildings are “nearly zero energy buildings” from 1 January 2030, and for new buildings occupied or owned by public authorities from 1 January 2028 [10]. The directive also requires EU countries to develop building renovation strategies so that existing buildings can be cost-effectively transformed into nearly zero-energy buildings. In this context, the construction sector should strive to use ecological materials, which at the same time make it possible to increase the energy efficiency of buildings and reduce heat losses, as well as to look for new energy saving opportunities and technological solutions to achieve almost zero CO_2_ emissions. According to the authors of works [11,12], the correct selection of building materials becomes a priority issue due to the inevitable increase in expenditure on heating buildings and the effects of climate change in the world. In order to determine the most energy-efficient and ecological material and construction solutions, extensive experimental research and numerical analyses should be carried out.

Works [7,13] emphasize that in order to achieve the high energy efficiency of buildings, it is necessary to focus on the causes of heat loss, especially in housing construction, and to implement efficient energy-saving technologies. Therefore, numerous studies are performed on the reduction of heat losses resulting from leaks in insulating systems and the occurrence of thermal bridges [14,15,16,17]. Kim et al. [18] state that as much as 50% of external building envelopes have thermal bridges, which, according to Ge et al. [19] increases the annual costs of space heating in the range of 38–42% and the annual space cooling costs by 8–26%. Shin et al. [20] add that the costs of space heating/cooling are usually underestimated, because usually, for ease of analysis, an ideal coating with one-dimensional heat flow is assumed, which does not reflect the presence of thermal bridges. In addition to obvious heat losses, thermal bridges are the cause of water vapor condensation and, consequently, the occurrence of mould fungi, which over time damages the construction and materials of building partitions. The authors of work [21] emphasize that the costs incurred to insulate external walls are compensated by savings during the operation of the building and that the use of thermal insulation is the easiest way to reduce heat loss by conduction. They add that convection and radiation should also be taken into account. Due to the complexity of the phenomenon of heat exchange in buildings, tools based on artificial intelligence are increasingly used in analyses. In work [22], deep learning algorithms for heat loss damage classification in buildings were used, and in work [23], the authors used neural networks to quickly and accurately predict the thermal distribution and heat loss of building structures.

One of the places in the building envelope where there are thermal bridges that require detailed analysis is the connection between the window and the external wall. The formation of thermal bridges at the window-to-wall interface results mainly from the fact that structural elements made of materials with different values of thermal conductivity coefficient are in contact there. In almost every such connection, the sealing material is polyurethane mounting foam. Therefore, Hallik et al. [24] undertook the analysis of air flow through joints filled with polyurethane foam. In the summary of the work, they stated that the use of assembly foam does not ensure the connections’ tightness against air penetration and the foam deteriorates over time. Therefore, various research and development centres conduct intensive research into the selection of materials and construction and technological solutions limiting thermal bridges [25,26]. The influence of the thickness of insulation layers [27,28] and moisture penetration on the thermal properties of building partitions was analysed in [29,30,31]. The possibilities of using recycled materials and bio-waste to improve the thermal parameters of external walls were analysed in [32,33]. In paper [34], the thermal-humidity and strength parameters of composite elements used during window installation were analysed, and in papers [35,36], additionally, the possibility of reinforcing composite beams with metal profiles was analysed. Many works, including [37,38], concern the improvement of the insulation parameters of the windows themselves. In papers [39,40], the influence of the window frame material was examined, and in paper [41], the influence of the method of filling the space between the panes on the reduction of heat loss was investigated. In [42], the influence of the thermal modernization of windows on the transmission of heat losses was analysed. Arici et al. [43] showed that replacing a double-glazed window with a triple-glazed one saves 50% of the heating energy, and the use of a four-glazed window results in as much as 67% savings.

The latest analyses of the impact of the size and shape of windows and their location on the energy efficiency of buildings and the reduction of CO_2_ emissions [44,45] clearly indicate that the size of the window is closely related to the energy consumption for heating or cooling the building and its lighting. Hafiz and Mhatre [46] add that daylight not only reduces the need for artificial lighting, but also significantly improves the well-being of users. The size of the window affects the area where the thermal bridge occurs and, thus, the amount of heat lost.

Experimental research in thermal chambers and numerical analyses indicate that the installation of windows in the thermal insulation layer is optimal due to the linear heat transfer coefficient, and thus results in a reduction in heat loss. Cappelletti et al. [47] draw attention to the fact that when determining the energy efficiency of buildings, apart from the type of components and materials from which the building structures are made of, the place and way in which these components (window, wall) are connected are important. The performed analyses showed that moving the window towards the insulation layer, depending on the method of insulating the reveal, reduces the value of the linear heat transfer coefficient *Ψ* by 58 ÷ 75%. Pawłowski and Krajewska [48] emphasize the importance of taking into account thermal bridges around the window, resulting from the method of installation, in the calculations of the energy efficiency of buildings. They noticed that even if the adopted system of material layers for the external partition meets the requirements for thermal insulation *U_c_ ≤ U_c(max)_* for external walls, after taking into account additional heat losses resulting from the presence of thermal bridges, the value of the coefficient *U_k_(U_2D_)* (heat transfer coefficient taking into account linear thermal bridges) increases by up to 35%. The authors also indicate that the method of insulating the window frame is important in the installation of windows. They showed that the insulation “overlapping” the frame, compared to the uninsulated frame, reduces the coefficient *Ψ* by about 20%, while the minimum temperature at the surface of the internal wall in the place of the thermal bridge increases by about 2 °C.

These studies are very important due to the possibility of improving the energy efficiency of buildings. Investors and building users often only pay attention to the time it takes to pay off the investment costs. In the case of innovative methods of window installation, such as the so-called warm window installation, the payback period often seems too distant and not worth incurring relatively high one-time expenses. However, due to the drastic change in the situation on the global energy market and new energy performance standards aimed at decarbonising the construction sector, reducing energy consumption and rationalizing energy use becomes a necessity. Saving energy necessary for heating or cooling buildings, among others thanks to the use of energy-saving and ecological building materials, is not only motivated by the care for the environment and the reduction of the greenhouse effect, but also by the limited supply of non-renewable energy resources. That is why the authors of this work took the effort to eliminate thermal bridges at the window-to-wall interface.

The authors’ research to date shows that the energy efficiency of buildings and heat losses resulting from the presence of thermal bridges at the window-to-wall interface are mainly influenced by three factors: the type of window installed, the method and place of window installation, and construction materials. The work is a continuation of research into the field of window installations in a layer of thermal insulation. In papers [34,36], the authors analysed the influence of the window type and the method of installation on the thermal and humidity parameters. The analyses showed that the value of the linear heat transmittance coefficient *Ψ* for the same variants of the external wall, but for windows with different heat transfer coefficients Uw, were very similar. The average difference between the values of the *Ψ* coefficient calculated for the window with the heat transfer coefficient *U_w_* = 0.9 W/(m^2^∙K) and *U_w_* = 0.6 W/(m^2^∙K) was about 0.0003 W/(m∙K). Experimental studies and numerical analyses confirmed that the installation of windows in thermal insulation is the most advantageous. In these works, the optimal shift of the window relative to the thermal insulation layer was determined based on the value of the *Ψ* coefficient. Similar aspects of heat flow in the steady state using the TRISCO program were analysed in [36,49,50,51]. The conclusions from the works [18,47,52] are consistent with the results of the authors’ work, although the analyses included different variants of window installation and different wall structures.

The aim of this work is to demonstrate the benefits resulting from the correct installation of the window, the appropriate location of the window in relation to the face of the external wall, as well as the correct selection of wall construction materials (bearing layer and insulating layer) and the thickness of these layers. Although many studies analysed the size of the linear thermal bridges at the window-to-wall interface depending on the structure and materials used for external walls, the influence of the thickness of individual layers of the external wall on this parameter was not investigated. Therefore, the authors of the study conducted numerical analyses of various external wall materials and different wall thicknesses, trying to determine which of these parameters has the greatest impact on the linear heat transfer coefficient *Ψ* for the adopted window installation method. In order to show the improvement in the energy efficiency of buildings, as a novelty, an analysis of the consumption of heating/cooling energy was carried out for the selected buildings. The thermal and humidity analyses were carried out using the TRISCO program, while the economic analysis was performed using the Audytor OZC program. It was found that the proposed system of window installation in the thermal insulation layer reduced the annual heating demand by at least 10% on average.

## 2. Installation of the Window in the Insulation Layer Using a Composite Frame

Elimination of thermal bridges in buildings is one of the problems faced by designers, investors, and future users of buildings. This issue is also important for users of existing buildings who want to reduce the costs of heating/cooling buildings, as well as those who want to ensure thermal comfort and those who care about the environment. A large number of windows in buildings is not only a fashion choice but are also utilized for energy considerations. On the one hand, in the heating season, heat gains resulting from solar radiation are included in the building’s energy balance as a significant source of heat. On the other hand, in the summer season, these profits are the reason for overheating of rooms and force the installation and activation of air conditioning systems. In addition, a greater number of windows in buildings is associated with an increased number of linear and point bridges at the window-to-wall interface. Therefore, the article analyses the influence of material parameters of the external wall on the size of thermal bridges.

### 2.1. Criteria for Evaluating the Quality of Window Installation

The linear (*Ψ*) and point (*χ*) heat transmittance coefficients are the basic parameters characterizing thermal bridges. Their values depend, among others, on the location of the thermal bridge, the materials from which the structural joints were made, and the quality of its execution. Knowing the values of these coefficients allows for assessing the impact of thermal bridges on heat loss through individual building elements. The coefficient values are calculated on the basis of the ISO 10211:2017 standard [53] or adopted on the basis of the values provided in the ISO 14683:2017 standard [54] or in the catalogues of thermal bridges [55,56]. Pawłowski [49], Martin et al. [57], or Viot et al. [58] encourage the use of numerical calculations for each newly introduced solution as catalogued or analytically and numerically calculated thermal bridges may differ significantly. The authors of work [59] estimated that the accuracy of thermal bridge calculation methods is ±5% for numerical calculations, ±20% for thermal bridge catalogues, ±20% for analytical calculations, and within 0 ÷ 50% for the values given in the ISO 14683:2017 [54].

The influence of selected wall materials of the wall and the analysed method of window installation on the thermal and humidity properties of the window-to-wall interface were assessed in terms of:

- Values of the corrected heat transfer coefficient *U_c_* for the external wall;
- Values of the linear heat transmittance coefficient *Ψ*;
- Temperature factor at the internal surface *f_Rsi,_,* the so called hygiene criterion; one of the certification criteria required by the Passive House Institute specified in the document [60];
- Increase in the heat transfer coefficient Δ*U_w(installed)_*, the so-called window installation efficiency criterion.

The corrected heat transfer coefficient *U_c_*, taking into account corrections resulting from leaks in the insulation layer and the use of mechanical fasteners that usually penetrate the insulation layer, was calculated in accordance with the ISO 6946:2017 standard [61] and compared with the requirements of European standards [62], as well as the requirements of The Passive House Institute (PHI) [60]. According to them, all newly erected buildings should be energy saving, i.e., the *U_c_* coefficient for external walls cannot be higher than the permissible value of the *U_c(max)_* coefficient, which, in accordance with the Polish law [63], is 0.2 W/(m^2^·K); and to meet the passive house standard, the heat transfer coefficient *U_c_* of the building envelope for a cool temperature climate zone should be less than *U_c(max)_* = 0.15 W/(m^2^·K) [64].

The values of the linear heat transmittance coefficient *Ψ* for all calculation variants were determined in accordance with the ISO 10211:2017 standard [53], taking into account the variable cross-section of the external wall with the window installed, which affects the calculation accuracy and is recommended in the ISO 12831-1:2017 standard [65]. According to The Passive House Institute, the requirement of a building free of thermal bridges for buildings with standard geometry is met when [60]:

(1) Ψ≤0.01 W/(m·K)

The guidelines of The Passive House Institute [60] and the ISO 13788:2013 standard [66] indicate the need to check whether there is a risk of mould growth in the area of thermal bridges (hygiene criterion). For this purpose, the following condition was checked [66]:

(2) $$f_{Rsi}\geq f_{Rsi(crit)}$$

where: *f_Rsi_*—temperature factor at the internal surface [–],

*f_Rsi(crit_*)—critical value of the temperature factor at the internal surface [–].

The critical value of the temperature factor *f_Rsi(crit_*_)_ can be determined in three ways:

1. Simplified—assuming room temperature *T_i_* = 20 °C and relative air humidity in the room *φ_i_* = 50%, *f_Rsi(crit)_* = 0.72;
2. Accurate—according to the procedure described in the ISO 13788:2013 standard [66];
3. Compliant with the requirements of The Passive House Institute [60] for a selected region of the world—in the case of Poland *f_Rsi(crit)_* = 0.75.

The quality of window installation was also confirmed by the efficiency criterion, i.e., the parameter Δ*U_w(installed)_* expressing the increase in the value of the heat transfer coefficient in the area of the thermal bridges at the window-to-wall interface. According to the guidelines of the Passive House Institute, it was calculated using the following formula [60]:

(3) $$\Delta U_{w(installed)}=\frac{\sum \psi_{install,i}\cdot l_{install,i}}{A_{w}}$$

where: Δ*U_w(installed_*_)_—increase in the heat transfer coefficient depending on the window installation [W/(m^2^·K)],

*Ψ_install,i_*—linear heat transmittance coefficient for a given installation situation [W/(m∙K)],

*l_install,i_*—length of the thermal bridge for a given installation situation [m],

*A_w_*—window surface [m²]—calculations were performed for the window with dimensions: 1.23 × 1.48 m.

The Passive House Institute recommends adopting the efficiency criterion for the heat transfer coefficient Δ*U_w(installed)_* = 0.05 W/(m^2^·K).

### 2.2. Numerical Models

**Figure 1:**
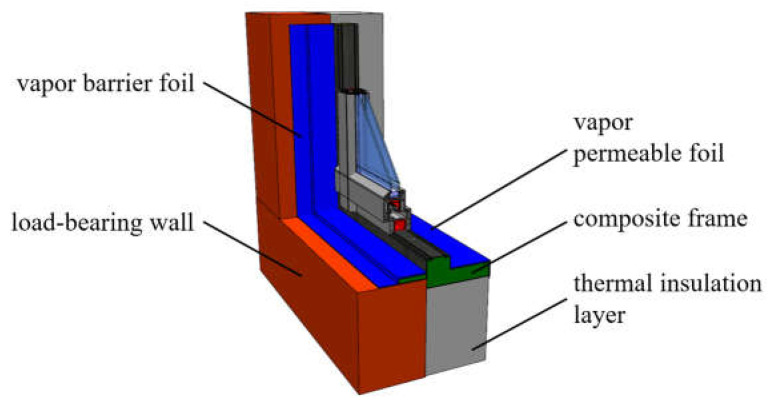
Analysed window installation system.

Based on the results of previous experimental and numerical studies, as well as on the literature analysis and a review of current window installation systems, the present investigations were carried out for the window installation system in the thermal insulation layer using a mounting composite frame, presented in Figure 1.

In order to evaluate this system and to determine the relationship between the selected geometrical and material parameters of the window-to-wall interface, calculations were carried out for various variants, in which the following assumptions were made:

- Three commonly used types of materials of the load-bearing layer of a double-layer external wall with a thermal conductivity coefficient:
- *λ* = 0.22 W/(m∙K)—aerated concrete blocks,
- *λ* = 0.313 W/(m∙K)—ceramic bricks,
- *λ* = 0.46 W/(m∙K)—silicate blocks,
- with a thickness of 240 mm, 300 mm and 360 mm;
- Three commonly used materials for the thermal insulation layer of the wall with the thermal conductivity coefficient:
- λ = 0.036 W/(m∙K)—expanded polystyrene,
- λ = 0.039 W/(m∙K)—mineral wool,
- λ = 0.021 W/(m∙K)—resol hard foam boards;
- The thickness of the thermal insulation layer in the range: from 150 mm to 300 mm for styrofoam and mineral wool, and from 90 to 200 mm for resol hard foam boards;
- The heat transfer coefficient of the window: *U_w_* = 0.6 W/(m^2^∙K) as for passive window on the basis of which thermal conductivity coefficient *λ* = 0.060 W/(m∙K) was calculated;
- The heat transfer coefficient of the composite assembly frame: *λ* = 0.02 W/(m∙K),
- Two window locations:
- First location: window flush with the face of the load-bearing layer,
- Second location: window optimally shifted into the thermal insulation layer, in accordance with the previously performed calculations [36].

The material properties of the load-bearing layer of the wall and the insulation layer were adopted in accordance with the technical catalogues provided by the manufacturers of these materials.

The thermal conductivity coefficient of the composite from which the mounting frame was made was determined experimentally in the HFM 446 Lambda Series–Heat Flow Meter for Testing Insulation Materials device.

**Figure 2:**
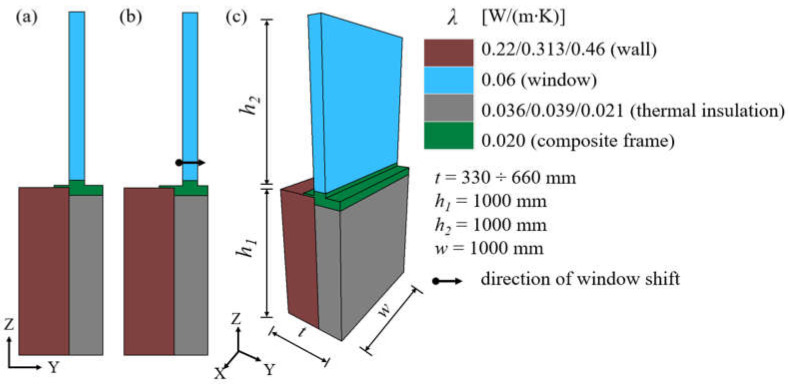
Geometric model and material properties: (**a**) window flush aligned with the face of the wall, (**b**) window shifted into the thermal insulation layer, (**c**) dimensions of the model.

In total, calculations were made for over 400 variants of an external wall with a window installed in two positions. Dimensions, geometry, and material properties for the analysed variants are shown in Figure 2.

**Figure 3:**
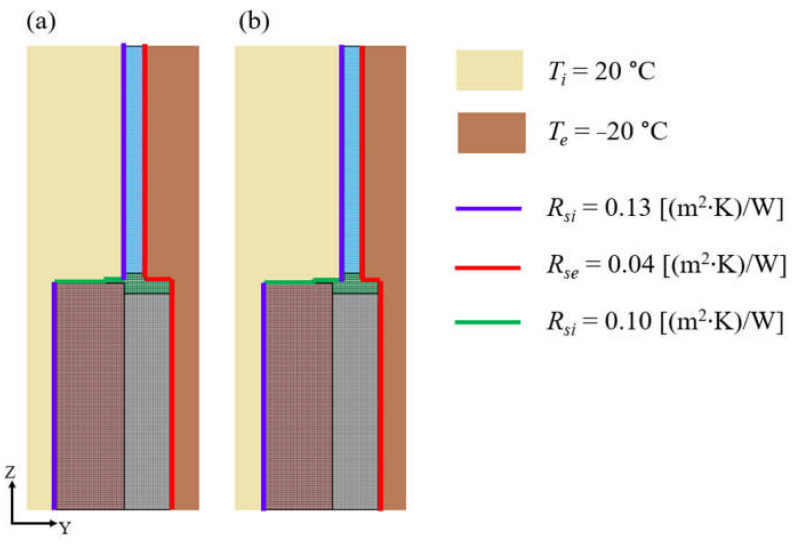
Numerical model and assumed boundary conditions: (**a**) window flush aligned with the face of the wall, (**b**) window shifted into the thermal insulation layer.

Numerical models were developed for all variants of the wall (Figure 3). Three-dimensional numerical analyses of heat flow were carried out using the TRISCO program, which is one of the fastest and most complete digital tools for the thermal analysis of building elements. It has geometric modelling functions and an optimized finite element solver. It also has a good graphical representation of isotherms. As the software developer TRISCO version 12.0w Physibel from Belgium [67] assures, the program is optimized for automatic use in accordance with EN ISO 6946 [61] and EN ISO 10211 [53]. The objects are modelled using rectangular blocks, with a minimum number of blocks required for superposition in space. It is possible to enter 2D AutoCAD drawings via a DXF file. Material properties (thermal conductivity, surface emissivity) are assigned to colours. Calculations of equivalent thermal conductivity for air voids are modelled as materials automatically. Boundary conditions are assigned to the surface at the boundary of the material with the surroundings or between materials, volume boundary conditions are assigned to material volume with constant temperature or heat power density, point boundary conditions are described by constant temperature or power.

The system generates a rectangular mesh of nodes passing through object block sur-faces. It has grid refinement functions. A solution is obtained through iteration and the number of nodes is only limited by the installed computer memory.

The following boundary conditions were assumed in the calculations: heat transfer resistance *R_si_* = 0.13 and *R_se_* = 0.04 (m^2^∙K)/W for horizontal heat flow and *R_si_* = 0.10 (m^2^∙K)/W for upward heat flow. It was assumed that the internal and external temperatures are constant and amount to *T_i_* = 20 °C and *T_e_* = −20 °C, respectively. The wall, the thermal insulation layer, and the composite assembly frame were modelled as rectangular elements. The cross-section of the window profile has been simplified by modelling it as a rectangular panel. A grid of finite elements with a uniform side equal to 1 mm was adopted. According to the software documentation, the calculating parameters were as follows:

  maximum number of iterations—10,000,
  absolute error in calculated temperatures—0.0001 °C,
  absolute error in calculated heat fluxes in the connector—0.001.

After the input of geometry and thermal properties, a system of linear equations was calculated based on the energy balance technique and solved using a fast iterative method. After performing the calculations, a digital result is created, which includes, among others: temperature and heat fluxes of the analysed model.

The proposed numerical model developed in the TRISCO program allows for a simple and accurate analysis of heat flow using a three-dimensional numerical method, which is emphasized as a great advantage of this program by the authors of works such as [50,51]. The adopted model does not allow for the analysis of elements other than rectangular ones, which, however, is not a major problem in the case of analysing the heat flow through the external wall with a window. The analysis of large spatial models with a generated mesh of finite elements with a small side value (e.g., 1 mm) involves an increase in computational time. The numerical models analysed in this article can also be used to investigate heat flow in transient thermal simulations.

### 2.3. Experimental Verification of Numerical Model

**Figure 4:**
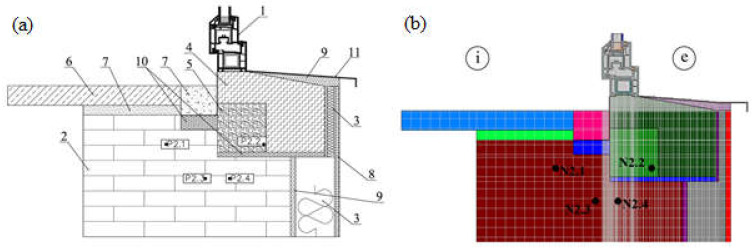
Model of the test stand: (**a**) location of measurement points in cross-section of real wall with window: P2.1–P2.4, (**b**) location of nodes N2.1–N2.4 in the numerical model; i—internal, e—external, 1—window, 2—structural wall, 3—thermal insulation, 4—insulating core, 5—sill beam, 6—internal window sill, 7—mortar, 8—external plaster, 9—glue, 10—mounting foam, 11—external window sill.

Before analysing the criteria for assessing the quality of window installation, the numerical model was verified based on a comparison of the results of numerical calculations and the results of experimental tests. The numerical model developed in the TRISCO program corresponded to the model of the actual test stand. Both the test stand (i.e., an external wall with a window installed) and the experiment were described in detail in [36]. During the measurement period lasting 28 days, the external temperature and the temperature at the selected points of the test stand were measured. The tests were conducted in autumn, winter, and spring. The arrangement of measurement points in the cross-section of the external wall is shown in Figure 4a, while the numerical model is presented in Figure 4b.

The process of heat flow between the interior of a room with a constant temperature of 20 °C and the external environment with varying temperature was analysed. The variable temperature was determined as a function of the change in actual temperature over time. For each measurement day, 24 external temperature measurements were taken, which gave 672 values. A grid of finite elements with a uniform side equal to 1 mm was adopted. A heat transfer resistance *R_si_* = 0.13 m^2^·K/W on the inner side of the wall and R*_se_* = 0.04 m^2^·K/W on the outer side for the horizontal direction of the heat flow were assumed.

The location of the N2.1–N2.4 nodes in the numerical models corresponds to the location of the measurement points in the real models.

**Figure 5:**
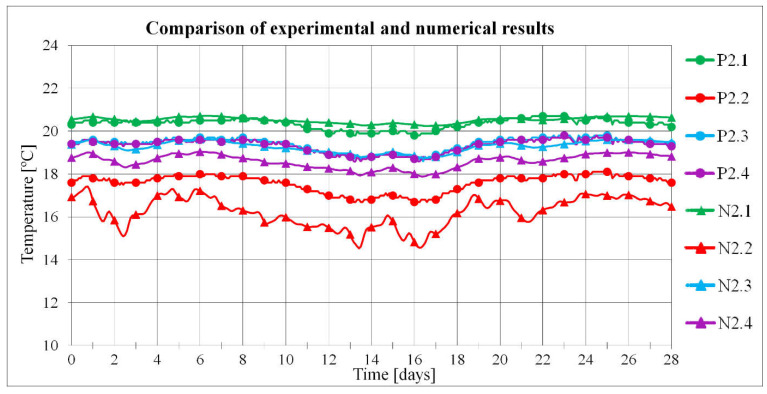
Comparison of experimental and numerical results.

A comparison of the temperatures measured with thermocouples at the test stand in autumn and the temperatures obtained as a result of numerical calculations is shown in Figure 5.

The actual temperature values turned out to be slightly higher compared to the numerically calculated values. For points P2.1 and N2.1 and for P2.3 and N2.3, the difference between the actual and numerical temperature values was on average 0.15 °C, and for points P2.4 and N2.4 was 0.7 °C. The largest temperature difference of 1.4 °C was obtained for points P2.2 and N2.2. This is due to the location of the measurement point and the numerical node in the composite mounting beam, where there is a high density of isotherms, making it difficult to read the temperature correctly. The average difference in temperature values over the entire measurement period between the experimental and numerical results was 0.7 °C.

Similar results were obtained for tests conducted in 28-day measurement periods during winter and spring. The average difference in temperature values in these measurement periods was 0.8 °C in winter and 0.7 °C in spring. Based on the obtained results, the correct operation of the numerical model and the validity of the adopted heat flow analysis method were confirmed.

## 3. Numerical Analysis—Results and Discussion

In order to verify whether the proposed window installation system in the external wall meets the criteria quality, the thermal and humidity parameters were analysed based on the results of numerical and analytical calculations.

When analysing the impact of the window installation method on thermal bridges, one cannot limit oneself only to window installation locations. It is also necessary to ensure adequate thermal insulation of the remaining surface of the external wall through the appropriate selection of material and thickness of the insulation layer so that the assumed requirements are met, e.g., for passive or energy-saving buildings. Table 1, Table 2 and Table 3 show the values of the *U_c_* coefficient calculated for the assumed external walls.

The obtained results of the *U_c_* coefficient show how important it is to choose the right materials for external walls. For the analysed variants of walls, commonly used construction and insulating materials in Europe and many countries around the world were adopted. It turns out that in 10% of cases, the requirements for walls in energy-saving buildings were not met and the maximum value *U_c(max)_* = 0.20 W/(m^2^∙K) was exceeded (the cases marked in red in Table 1, Table 2 and Table 3). Only 49% of the adopted external wall variants meet the requirements for passive houses, achieving a wall heat transfer coefficient lower than 0.15 W/(m^2^∙K) (the cases marked in both red and blue in Table 1, Table 2 and Table 3 do not meet the requirements for passive houses).

It follows that research into insulating materials should be developed and innovative solutions should be used, such as PIR foam insulation boards, vacuum panels, cellulose, or aerogels, which allow effective thermal insulation of the external wall without the need for very thick insulating layers. Thick thermal insulation means that less sunlight reaches the interior of the buildings and takes up more space. The thickness of the insulation layer is particularly important when insulating the walls from the inside of the room, where the priority is to minimize the loss of usable space. For example, a similar wall heat transfer coefficient *U_c_* is obtained for walls with an insulation layer of polystyrene or mineral wool with a thickness of 200 mm, as in the case of using resol hard foam boards but with a thickness of 120 mm.

The values of the linear heat transmittance coefficient *Ψ* were calculated for all variants of the external wall with windows at two positions: (1) window flush aligned with the face of the wall, and (2) window shifted into the thermal insulation layer to the distance ensuring the lowest coefficient *Ψ*.

Analysing the influence of the load-bearing wall material, assuming the same insulating material, it was found that the lowest values of the linear heat transmittance coefficient *Ψ* in each of the analysed cases were obtained for the wall with the load-bearing layer made of silicate blocks with a thickness of 24 cm, i.e., the material with the highest thermal conductivity coefficient *λ*. The better (lower) the thermal conductivity coefficient of the load-bearing layer of the wall, the worse (higher) the value of the *Ψ* coefficient, regardless of the type and thickness of the material and thermal insulation layer. However, the greater the thickness of the load-bearing layer of the wall, the smaller the differences between the values of the *Ψ* coefficient calculated for the thermal insulation layer with a thickness of 150 mm and 300 mm.

The material of the insulating layer has a greater impact on the values of the linear heat transmittance coefficient for the adopted method of window installation. Assuming the same material and thickness of the load-bearing layer, it turns out that the lower the thermal conductivity coefficient of the insulating material, the lower the values of the linear heat transmittance coefficient. While for variants with expanded polystyrene and mineral wool similar results were obtained for individual cases, definitely lower values were obtained for walls insulated with resol hard foam boards.

**Figure 6:**
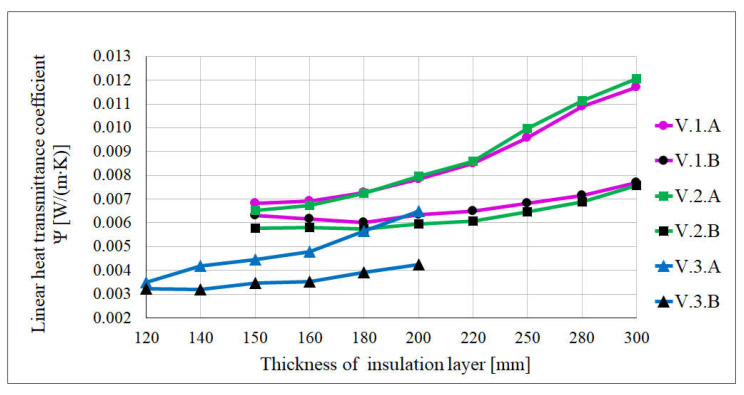
Change of the linear heat transmittance coefficient *Ψ*.

- The value of the *Ψ* coefficient is also affected by the location of the window. In variants with the window flush aligned with the face of the wall (location A in Figure 6), the lowest values were obtained for the smallest thickness of the insulation layer, and with the increase of the thickness of the insulating layer, the values of the coefficient *Ψ* increase. However, in cases where the window was moved into the insulating layer (location B in Figure 6), the values of the linear heat transmittance coefficient were very similar to each other, regardless of the thickness of the insulating layer. The change in the linear heat transmittance coefficient depending on the thickness of the insulation layer for the aforementioned window locations for the following three wall variants is shown in Figure 6: V1—the wall made of 240 mm thick aerated concrete blocks and expanded polystyrene as an insulating layer (V.1.A and V.1.B);
- V2—the wall made of 240 mm thick ceramic airbricks and mineral wool as an insulating layer (V.2.A and V.2.B);
- V3—the wall made of 240 mm thick silicate blocks and resol hard foam boards as an insulating layer (V.3.A and V.3.B).

Due to the possibility of thermal bridges around the window, it is more advantageous to use wall variants with higher thermal conductivity coefficients for the structural layer and smaller thicknesses of the insulation layer. However, due to the fact that in most cases such arrangement of the load-bearing and insulating layers does not meet the requirement of adequate thermal insulation of the wall *U_c_*, this, consequently, causes greater heat losses in the entire building than those resulting from linear bridges around the window.

The requirement of a building free of thermal bridges with the coefficient *Ψ* ≤ 0.01 W/(m∙K) (in accordance with the guidelines of the Passive House Institute for a standard building geometry [60]) was met by as many as 84% of the analysed variants, including all variants with resol hard foam boards, regardless of the material used for the load-bearing layer. After excluding the variants for which the external wall did not meet the thermal insulation requirement for the wall of the energy-saving building, the number of variants decreases from 414 to 366, among which 81% achieved the coefficient *Ψ* ≤ 0.01 W/(m∙K). If we additionally take into account the requirements for the thermal insulation of the passive building wall, the number of variants is reduced to 210, of which 75% achieve a linear heat transmittance coefficient below 0.01 W/(m∙K).

**Figure 7:**
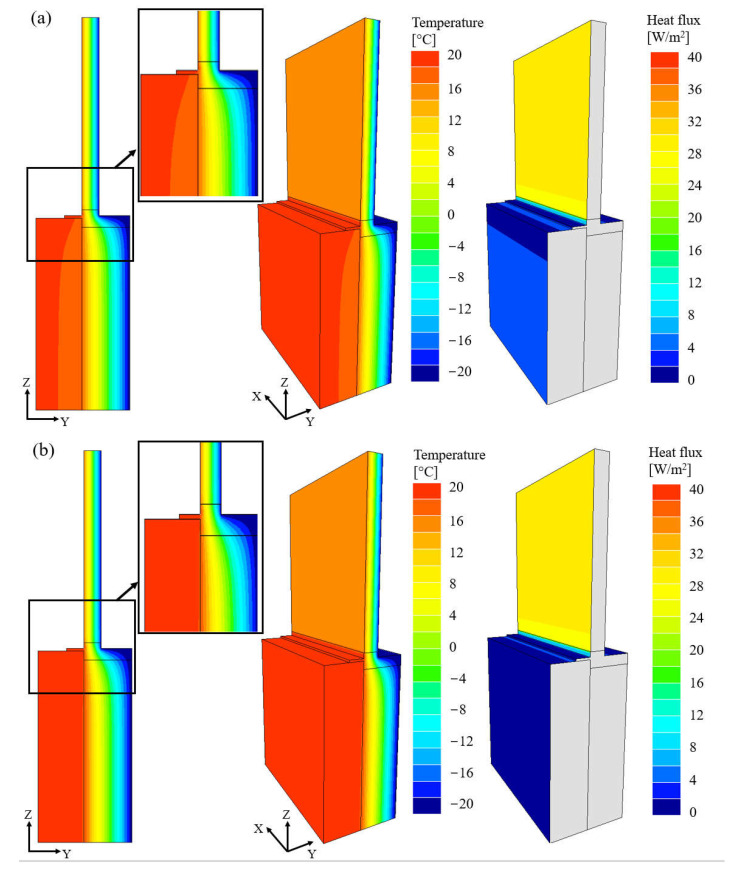
Temperature distribution and heat flux lines for wall made of silicate blocks and a 200 mm thick insulating layer made of: (**a**) expanded polystyrene, (**b**) resol hard foam boards.

The results of numerical simulations for the wall variants with a structural layer made of silicate blocks and a layer of thermal insulation made of polystyrene or resol hard foam board with thickness of 200 mm, as well as a window with a coefficient *U_w_* = 0.6 W/(m^2^∙K), are shown in Figure 7. The temperature distribution (isotherms) in the wall section, temperature distribution with axonometric view on the inside of the wall, and distribution of the heat flow lines (adiabats) are presented from left to right.

The analysis of the results showed that the location of the window in the wall affects the position of the isotherms in the entire external partition. Areas of non-linear heat flow with irregular isotherms are relatively small. Despite the difference in the values of the thermal conductivity coefficient *λ* for polystyrene and mineral wool, the course of isotherms and the distribution of heat fluxes are similar; therefore, the results are presented only for polystyrene (Figure 7a). Differences are noticeable for the variant of the wall with resol hard foam boards, where a constant temperature is maintained throughout the cross-section of the load-bearing layer and the flow of heat fluxes is close to zero (Figure 7b).

For all analysed variants, it was checked whether there was a risk of mould growth in the area of thermal bridges, i.e., the hygiene criterion. The value of the temperature coefficient *f_Rsi_* was calculated. It was found that condition (2) was met in each variant, both at the critical value of the temperature coefficient *f_Rsi(crit)_* equal to 0.75 and 0.72. It should be emphasized that around the window installed with the use of a composite frame, there are no places with significantly reduced temperature or large temperature differences in the corners of the window, which could lead to condensation of water vapor or the development of mould fungi.

In accordance with the guidelines of the Passive House Institute [60], the window installation efficiency criterion, i.e., the increase in the value of the heat transfer coefficient *ΔU_w(installed)_*, was also checked. All variants were calculated for the total window construction (lower section + side section + upper section = one installation case) for windows with dimensions of 1.23 × 1.48 m and heat transfer coefficient *U_w_* = 0.6 W/(m^2^·K). In each analysed variant, the calculated values of Δ*U_w(installed)_* were lower than the recommended value of 0.05 W/(m^2^ K). On average, this coefficient was 0.025 W/(m^2^ K) for walls with expanded polystyrene and wool, and 0.016 W/(m2 K) for walls with resol hard foam boards. The increase in the heat transfer coefficient of the window after installation in relation to the unmounted window was within the range of 2.4 ÷ 6.3% for walls with expanded polystyrene and wool, and within the range of 1.6 ÷ 3.7% for walls with resol hard foam boards.

All variants of the external wall with a window installed in the thermal insulation layer analysed in the article were checked for meeting the requirements of four criteria:

– Values of the corrected heat transfer coefficient *U_c_* for the external wall;
– Values of the linear heat transmittance coefficient *Ψ*;
– Temperature factor at the internal surface *f_Rs_*_i_ (the so-called hygiene criterion);
– Increase in the heat transfer coefficient Δ*U_w(installed)_* (the so-called window installation efficiency criterion).

In many cases, a given installation variant met one or two of the four criteria. In order to systematize the analysis results, Table 4 was prepared, from which it is possible to read which variants meet the particular criteria recommended by the Passive House Institute [60], the ISO 13788 standard [66], and the regulation [63].

From Table 4, it is easy to see what effect a change of, e.g., the thickness of the insulating layer, causes.

Among the 414 analysed variants of the external wall (bearing layer + thermal insulation), all four assembly quality assessment criteria were met simultaneously by 297 variants in the case of energy-saving buildings, and only 153 variants in the case of passive houses.

## 4. Impact of Window Installation Method on Energy Efficiency of Buildings

In order to demonstrate the impact of the proposed window installation system on improving the energy efficiency of buildings, the Audytor OZC program [68] was used. Generally, the program is used to support the building heat load calculations and to determine the annual demand for the heating and cooling of buildings. The annual demand for thermal energy was determined for each building. For traditional window installation, the analyses were carried out assuming a standard value of the linear heat transmittance coefficient in the frame *Ψ*_1_ = 0.2 W/(m·K), while in the case of the analysed system of window installation in the insulation layer using a composite mounting frame, the calculated value *Ψ*_2_ = 0.007 W/(m·K) was used. After entering the data on the buildings selected for analysis into the Audytor OZC program, the analysis was carried out for the following buildings:

- Single-family residential building;
- Multi-family residential building;
- Public utility building—kindergarten.

The characteristics of individual buildings are presented in Table 5.

In order to demonstrate the reduction in heat energy demand due to the installation of the window with the use of the proposed composite mounting frame, whose *Ψ*_2_ = 0.007 W/(m K), calculations of the annual heating demand *Q_H,nd_* were performed in accordance with [69,70].

Table 6 presents the calculated values of the design transmission heat loss and the annual heating demand in the analysed buildings for both values of the coefficient *Ψ*.

Analysing the obtained values of the annual heating demand in the analysed buildings, it is possible to notice the impact of changing the window installation method on the reduction of transmission heat loss. The use of the window installation system in the thermal insulation layer with the use of a composite mounting frame reduced the demand for heat energy by 15.4% for a single-family building, by 11.7% for a multi-family building, and by 12.3% for a kindergarten building. It should be emphasized that not only will the heating costs decrease, but also the emission of greenhouse gases into the atmosphere will decrease thanks to the reduced demand for energy resources.

## 5. Conclusions

Based on the literature review and the carried-out analysis of the numerical calculations, it was found that:

- The window installation method and the type of wall structural materials are interrelated and should be considered simultaneously;
- The type of material of the insulating layer and its thickness have a dominant impact on meeting the adopted criteria for assessing the quality of window installation and applicable standard requirements;
- The right choice of materials for both the load-bearing layer and the insulating layer allows for a significant reduction in heat loss through penetration, and thus to improve the energy efficiency of buildings;
- The correct selection of a window installation system and wall structural materials allows for reduction in the amount of energy needed to heat/cool buildings, and thus reduce heating/cooling costs, as well as reduce greenhouse gas emissions;
- The system of window installation in the thermal insulation layer allowed to reduce the annual heating demand by at least 10% on average;
- Out of 414 analysed variants of the external wall (load-bearing layer + thermal insulation), all required criteria for assessing the quality of window installation (corrected heat transfer coefficient *U_c_* for the external wall, linear heat transmittance coefficient *Ψ*, temperature factor at the internal surface *f_Rsi_*, increase in the heat transfer coefficient Δ*U_w(installed)_*) were met simultaneously by 297 variants in the case of energy-efficient buildings and only 153 variants in the case of passive houses.

The presented analyses concern only one method of installing a window in the thermal insulation layer. It should be checked whether similar relationships between external wall materials and the linear heat transfer coefficient occur for other window installation methods or other types of walls (e.g., with internal insulation or three-layer walls), which will be the subject of further analyses. In addition, the possibility of using ecological and recycled materials as insulation materials for external walls will be explored.

## Figures and Tables

**Table 1:** *U_c_* coefficient for external wall with insulating layer made of expanded polystyrene.

Heat Transfer Coefficient *U_c_* [W/(m^2^∙K)] Expanded Polystyrene (*λ* = 0.036 W/(m∙K))									
Layer Thickness [mm]		150	160	180	200	220	250	280	300
Aerated concrete block *λ* = 0.220 W/(m∙K)	240	0.197 **	0.187 **	0.171 **	0.157 **	0.146	0.131	0.119	0.112
	300	0.187 **	0.178 **	0.163 **	0.151 **	0.140	0.127	0.115	0.109
	360	0.178 **	0.170 **	0.157 **	0.145	0.135	0.122	0.112	0.106
Ceramic block *λ* = 0.313 W/(m∙K)	240	0.210 *	0.199 **	0.181 **	0.166 **	0.153 **	0.137	0.124	0.116
	300	0.202 *	0.192 **	0.175 **	0.161 **	0.148	0.133	0.121	0.114
	360	0.195 **	0.185 **	0.169 **	0.156 **	0.144	0.130	0.118	0.112
Silicate block *λ* = 0.460 W/(m∙K)	240	0.222 *	0.210 *	0.189 **	0.173 **	0.159 **	0.142	0.128	0.120
	300	0.215 *	0.204 *	0.185 **	0.169 **	0.156 **	0.139	0.126	0.118
	360	0.209 *	0.199 **	0.181 **	0.165 **	0.152 **	0.137	0.124	0.116

* values do not meet the requirements for walls in energy-saving buildings; ** values do not meet the requirements for walls in passive houses.

**Table 2:** *U_c_* coefficient for external wall with insulating layer made of mineral wool.

Heat Transfer Coefficient *U_c_* [W/(m^2^∙K)] Mineral Wool (*λ* = 0.039 W/(m∙K))									
Layer Thickness [mm]		150	160	180	200	220	250	280	300
Aerated concrete block *λ* = 0.220 W/(m∙K)	240	0.208 *	0.198 **	0.181 **	0.167 **	0.155 **	0.139	0.127	0.120
	300	0.197 **	0.188 **	0.173 **	0.160 **	0.148	0.134	0.123	0.116
	360	0.187 **	0.179 **	0.165 **	0.153 **	0.143	0.129	0.119	0.112
Ceramic block *λ* = 0.313 W/(m∙K)	240	0.223 *	0.212 *	0.192 **	0.176 **	0.163 **	0.146	0.132	0.124
	300	0.214 *	0.203 *	0.186 **	0.171 **	0.158 **	0.142	0.129	0.121
	360	0.205 *	0.196 **	0.179 **	0.165 **	0.153 **	0.138	0.126	0.119
Silicate block *λ* = 0.460 W/(m∙K)	240	0.236 *	0.223 *	0.202 *	0.184 **	0.169 **	0.151 **	0.137	0.128
	300	0.229 *	0.217 *	0.197 **	0.180 **	0.166 **	0.148	0.134	0.126
	360	0.222 *	0.211 *	0.192 **	0.176 **	0.162 **	0.146	0.132	0.124

* values do not meet the requirements for walls in energy-saving buildings. ** values do not meet the requirements for walls in passive houses.

**Table 3:** *U_c_* coefficient for external wall with insulating layer made of resol hard foam boards.

Heat Transfer Coefficient *U_c_* [W/(m^2^∙K)] Resol Hard Foam Boards (*λ* = 0.021 W/(m∙K))									
Layer Thickness [mm]		90	100	120	140	150	160	180	200
Aerated concrete block *λ* = 0.220 W/(m∙K)	240	0.202 *	0.186 **	0.161 **	0.142	0.134	0.127	0.115	0.105
	300	0.191 **	0.177 **	0.155 **	0.137	0.130	0.123	0.112	0.102
	360	0.182 **	0.169 **	0.148	0.132	0.125	0.119	0.108	0.100
Ceramic block *λ* = 0.313 W/(m∙K)	240	0.215 *	0.198 **	0.170 **	0.149	0.140	0.133	0.120	0.109
	300	0.207 *	0.191 **	0.165 **	0.145	0.137	0.129	0.117	0.107
	360	0.199 **	0.184 **	0.160 **	0.141	0.133	0.126	0.114	0.105
Silicate block *λ* = 0.460 W/(m∙K)	240	0.227 *	0.208 *	0.177 **	0.155 **	0.145	0.137	0.123	0.112
	300	0.221 *	0.202 *	0.173 **	0.152 **	0.143	0.135	0.121	0.110
	360	0.215 *	0.197 **	0.170 **	0.149	0.140	0.132	0.119	0.109

* values do not meet the requirements for walls in energy-saving buildings. ** values do not meet the requirements for walls in passive houses.

**Table 4:** Selection of the window installation variant in the insulation layer depending on the adopted criterion.

Aerated concrete blocks		Expanded polystyrene								Mineral wool								Resol hard foam boards							
		150	160	180	200	220	250	280	300	150	160	180	200	220	250	280	300	90	100	120	140	150	160	180	200
240 mm	1’st location	X **	X **	X **	X **	X	X	X	X	X *	X **	X **	X **	X **	X	X	X	X *	X **	X **	X	X	X	X	X
	2’nd location	X **	X **	X **	X **	X	X	X	X	X *	X **	X **	X **	X **	X	X	X	—	—	X **	X	X	X	X	X
300 mm	1’st location	X **	X **	X **	X **	X	X	X	X	X **	X **	X **	X **	X	X	X	X	X **	X **	X **	X	X	X	X	X
	2’nd location	X **	X **	X **	X **	X	X	X	X	X **	X **	X **	X **	X	X	X	X	—	—	X **	X	X	X	X	X
360 mm	1’st location	X **	X **	X **	X	X	X	X	X	X **	X **	X **	X	X	X	X	X	X **	X **	X	X	X	X	X	X
	2’nd location	X **	X **	X **	X	X	X	X	X	X **	X **	X **	X	X	X	X	X	—	—	X	X	X	X	X	X
Ceramic blocks		Expanded polystyrene								Mineral wool								Resol hard foam boards							
		150	160	180	200	220	250	280	300	150	160	180	200	220	250	280	300	90	100	120	140	150	160	180	200
240 mm	1’st location	X *	X **	X **	X **	X **	X	X	X	X *	X *	X **	X **	X **	X	X	X	X *	X **	X **	X	X	X	X	X
	2’nd location	X *	X **	X **	X **	X **	X	X	X	X *	X *	X **	X **	X **	X	X	X	—	—	X **	X	X	X	X	X
300 mm	1’st location	X *	X **	X **	X **	X **	X	X	X	X *	X *	X **	X **	X **	X	X	X	X *	X **	X **	X	X	X	X	X
	2’nd location	X *	X **	X **	X **	X **	X	X	X	X *	X *	X **	X **	X **	X	X	X	—	—	X **	X	X	X	X	X
360 mm	1’st location	X **	X **	X **	X **	X	X	X	X	X *	X *	X *	X **	X **	X	X	X	X **	X **	X **	X	X	X	X	X
	2’nd location	X **	X **	X **	X **	X **	X	X	X	X *	X **	X **	X **	X **	X	X	X	—	—	X **	X	X	X	X	X
Silicate blocks		Expanded polystyrene								Mineral wool								Resol hard foam boards							
		150	160	180	200	220	250	280	300	150	160	180	200	220	250	280	300	90	100	120	140	150	160	180	200
240 mm	1’st location	X *	X *	X **	X **	X **	X	X	X	X *	X *	X *	X **	X **	X **	X	X	X *	X *	X **	X **	X	X	X	X
	2’nd location	X *	X *	X **	X **	X **	X	X	X	X *	X *	X *	X **	X **	X **	X	X	—	—	X **	X **	X	X	X	X
300 mm	1’st location	X *	X *	X **	X **	X **	X	X	X	X *	X *	X **	X **	X **	X	X	X	X *	X *	X **	X **	X	X	X	X
	2’nd location	X *	X *	X **	X **	X **	X	X	X	X *	X *	X **	X **	X **	X	X	X	—	—	X **	X **	X	X	X	X
360 mm	1’st location	X *	X **	X **	X **	X **	X	X	X	X *	X *	X **	X **	X **	X	X	X	X *	X **	X **	X	X	X	X	X
	2’nd location	X *	X **	X **	X **	X **	X	X	X	X *	X *	X **	X **	X **	X	X	X	—	—	X **	X	X	X	X	X

1st location: window flush aligned with the face of the load-bearing layer; 2nd location: window optimally shifted into the thermal insulation layer; *—do not meet the requirement for walls in energy-saving buildings; **—do not meet the requirement for walls in passive houses; X—meet the requirement for building free of thermal bridges with the coefficient *Ψ* ≤ 0.01 W/(m∙K); X—do not meet the requirement for building free of thermal bridges with the coefficient *Ψ* ≤ 0.01 W/(m∙K).

**Table 5:** Characteristics of buildings selected for economic analysis.

Building Type	Single-Family Residential Building	Multi-Family Residential Building	Kindergarten
Area of premises of building with regulated temperature *A_H_* [m^2^]	84.4	1974.2	16,643
Heated volume of rooms in building with adjustable temperature *V_H_* [m^3^]	289.8	4887.7	3886.6
Type of heating system in building	underfloor heating	convection heating	convection heating
Share of heat loss through windows [%]	13.6%	29.4%	9.4%
Heat transfer coefficient of windows *U_w_* [W/m^2^·K]	0.9	1.8	0.8
Area of windows in building *A_w_* [m^2^]	19.1	349.4	204.8
Sum of lengths on which there is linear thermal bridge [m]	50.42	1023.00	512.68
External wall construction	Porotherm brick 250 mm + styrofoam 150 mm	aerated concrete blocks 240 mm + styrofoam 40 mm + aerated concrete blocks 120 mm + styrofoam 100 mm	reinforced concrete 170 mm + styrofoam 50 mm + reinforced concrete 50 mm + styrofoam 140 mm
Heat transfer coefficient of outer wall *Uc* [W/(m^2^·K)]	0.198	0.184	0.197

**Table 6:** Calculation results for the analysed buildings.

	Single-Family Residential Building	Multi-Family Residential Building	Kindergarten
Linear heat transmittance coefficient	Design transmission heat loss *Φ_T_* [W]		
*Ψ_1_* = 0.200 W/(m·K)	4262	72,806	28,726
*Ψ_2_* = 0.007 W/(m·K)	3703	64,956	24,842
Difference	559	7850	3884
Linear heat transmittance coefficient	Annual heating demand *Q_H,nd_* [GJ/year]		
*Ψ_1_* = 0.200 W/(m·K)	26.23	515.80	223.85
*Ψ_2_* = 0.007 W/(m·K)	22.18	455.62	196.39
Difference	4.05	60.18	27.46
Linear heat transmittance coefficient	Annual heating demand *Q_H,nd_* [kWh/year]		
*Ψ_1_* = 0.200 W/(m·K)	7285	143,279	62,180
*Ψ_2_* = 0.007 W/(m·K)	6160	126,561	54,554
Difference	1125	16,718	7626

## Data Availability

The data presented in this study are available on request from the corresponding author.
